# Refining imaging tools to detect advanced fibrosis: could liver surface nodularity address an unmet need in the NAFLD epidemic?

**DOI:** 10.1007/s00330-021-08508-2

**Published:** 2022-01-27

**Authors:** Jörn M. Schattenberg, Tilman Emrich

**Affiliations:** 1 Department of Internal Medicine I, University Medical Center of the Johannes Gutenberg-University, Langenbeckstrasse 1, 55131 Mainz, Germany; 2 Metabolic Liver Research Program, University Medical Center of the Johannes Gutenberg-University, Mainz, Germany; 3 Division of Cardiovascular Imaging, Department of Radiology and Radiological Science, Medical University of South Carolina, Charleston, SC USA; 4 Department of Diagnostic and Interventional Radiology, University Medical Center of the Johannes Gutenberg-University, Mainz, Germany; 5 German Center for Cardiovascular Research (DZHK), Partner-Site Rhine-Main, Mainz, Germany

Dioguardi-Burgio et al [1] present a study that explores liver surface nodularity (LSN) on MRI to identify patients with advanced fibrosis related to non-alcoholic fatty liver disease (NAFLD). NAFLD is a highly prevalent liver disease with a high economic burden [2]. Still, the diagnosis rates are relatively low and even cirrhotic disease stages are only infrequently diagnosed. Today, there are multiple barriers that preclude the rapid identification of patients with advanced liver disease, even when they are under regular medical care, e.g., related to type 2 diabetes. Importantly, the mortality of patients living with NAFLD has been linked to the disease stages and excess mortality occurs in patients with advanced liver fibrosis stages defined as F3 and higher according to the NASH CRN staging classification [3].

In this retrospective cohort study at a tertiary care center in France, patients who underwent liver biopsy and received MRI as part of their clinical workup within 3 months were included. Data on 142 patients were available and 37% of these had advanced fibrosis defined as the histological disease stages F3 and F4. The AUC of LSN in this analysis was 0.838 [95CI 0.767–0.894]. These numbers are high, but there are important caveats that have to be considered when assessing diagnostic markers of liver fibrosis. The AUC is highly dependent on the quality of the reference standard—in this analysis liver biopsy—and confounders include the degree of fragmentation and the length of the biopsy [4]. Coming from expert centers, the highest quality can be assumed; however, aspects of the quality of biopsies are only infrequently reported [5]. The referral character of the involved centers impacts the pretest probability of the target condition and both the AUC [6] and the positive and negative predictive values will be different outside of this study population. Importantly, most non-invasive biomarkers for the assessment of liver fibrosis do not exceed 0.85 when compared to liver histology [7].

From a more general perspective, the use of MRI over CT appears favorable related to safety aspects, while in clinical care, many more patients do receive CT scans for a wide range of indications. The concept to use LSN to detect advanced liver fibrosis on CT scan has previously successfully shown for hepatic fibrosis of different indications [8], chronic viral hepatitis c [9], and portal hypertension [10]. Independent of the imaging modalities, it will be of great importance to report and acknowledge incidental hepatic steatosis on imaging. In the aftermath of the current analysis, it has to be considered to include LSN in radiology reports to improve the identification of asymptomatic at-risk patients with advanced liver disease. Therefore, training of both radiologists and physicians ordering radiological testing on the importance of NAFLD and more specifically the concept of advanced fibrosis as well as associated complications will be of great importance.

Currently, there are several non-invasive tests (NITs) being developed as diagnostic biomarkers to detect advanced fibrosis related to NAFLD [10]. Most current concepts aim at ruling out patients with a test that is widely available and comes at a low cost in an initial step, e.g., the FIB-4 score. Next, a more expensive test in an enriched population can be applied. This concept was also presented in the current study. By using LSN sequentially to FIB-4, 73% could be ruled out as advanced fibrosis, whereas a comparable number of cases were ruled in in the developmental cohort. While these numbers are acceptable, the use of imaging biomarker over blood-based biomarker carries the disadvantage of higher costs.

Beyond the use as a diagnostic biomarker, exploration of LSN as a prognostic biomarker seems appealing. In the realm of blood-based tests, recently the enhanced liver fibrosis (ELF) test received FDA approval. Looking at imaging modalities, magnetic resonance elastography (MRE) predicted the risk of future decompensation or death in cirrhosis with a 1-year probability of 9% with a baseline LSM above 5 kPa and of 20% with MRE above 8 kPa [11]. However, MRE is a technically challenging examination that requires dedicated equipment, training, and sequences. In contrast, LSN could be applied to any type of cross-sectional liver imaging.

The current study is remarkable for the following reasons. There is an urgent need to refine NITs and advance the field of diagnostic biomarkers [12]. As abdominal imaging (CT or MRI) is frequently performed, this could be one link to improve the diagnosis rates for a disease that is highly preventable but largely underdiagnosed. This will be of particular interest as the linkage to care will be further simplified with pharmacological options that are expected in 2023. On the other hand, the lack of awareness of NAFLD and hepatic fibrosis in most areas of the health sector and with health care providers—including radiologists—is an important aspect that needs to be addressed. Here, machine learning–supported image analysis tools could alert and direct physicians on findings that would then make it into a final report. The LSN analysis could be implemented as a simple test leading to the flagging of patients that qualify for additional workup. The data presented by Dioguardi-Burgio et al [1] expands and highlights the important role that MR scans can have in the future to detect a largely asymptomatic disease with a very high prevalence.

## Declarations

### Guarantor

The scientific guarantor of the editorial comment is Jörn M. Schattenberg, MD.

### Statistics and biometry

No complex statistical methods were necessary for this paper.

### Methodology

•Editorial Comment
